# Characterizing the invasion of different breast cancer cell lines with distinct *E-cadherin* status in 3D using a microfluidic system

**DOI:** 10.1007/s10544-019-0450-5

**Published:** 2019-11-23

**Authors:** H. Eslami Amirabadi, M. Tuerlings, A. Hollestelle, S. SahebAli, R. Luttge, C. C. van Donkelaar, J. W. M. Martens, J. M. J. den Toonder

**Affiliations:** 1 Microsystems group, Department of Mechanical Engineering and Institute for Complex Molecular Systems (ICMS), Eindhoven University of Technology, Groene Loper 15, 5612AZ Eindhoven, the Netherlands; 2 Healthy living division, TNO, Zeist, the Netherlands; 3 Institute for Pharmeceutical Sciences, Department of Pharmacology, Utrecht University, Utrecht, the Netherlands; 4 Orthopaedic Biomechanics group, Department of Biomedical Engineering and Institute for Complex Molecular Systems (ICMS), Eindhoven University of Technology, Groene Loper 15, 5612AZ Eindhoven, the Netherlands; 5 Department of Medical oncology, Erasmus MC Cancer Institute, Erasmus University Medical Center, Rotterdam, the Netherlands

**Keywords:** Cancer invasion, Breast cancer cell lines, Microfluidics, Chemotactic gradient, Invasion mode, E-cadherin

## Abstract

E-cadherin is a cell-cell adhesion protein that plays a prominent role in cancer invasion. Inactivation of *E-cadherin* in breast cancer can arise from gene promoter hypermethylation or genetic mutation. Depending on their *E-cadherin* status, breast cancer cells adopt different morphologies with distinct invasion modes. The tumor microenvironment (TME) can also affect the cell morphology and invasion mode. In this paper, we used a previously developed microfluidic system to quantify the three-dimensional invasion of breast cancer cells with different *E-cadherin* status, namely MCF-7, CAMA-1 and MDA-MB-231 with wild type, mutated and promoter hypermethylated *E-cadherin*, respectively. The cells migrated into a stable and reproducible microfibrous polycaprolactone mesh in the chip under a programmed stable chemotactic gradient. We observed that the MDA-MB-231 cells invaded the most, as single cells. MCF-7 cells collectively invaded into the matrix more than CAMA-1 cells, maintaining their E-cadherin expression. The CAMA-1 cells exhibited multicellular multifocal infiltration into the matrix. These results are consistent with what is seen in vivo in the cancer biology literature. In addition, comparison between complete serum and serum gradient conditions showed that the MDA-MB-231 cells invaded more under the serum gradient after one day, however this behavior was inverted after 3 days. The results showcase that the microfluidic system can be used to quantitatively assess the invasion behavior of cancer cells with different E-cadherin expression, for a longer period than conventional invasion models. In the future, it can be used to quantitatively investigate effects of matrix structure and cell treatments on cancer invasion.

**Electronic supplementary material:**

The online version of this article (10.1007/s10544-019-0450-5) contains supplementary material, which is available to authorized users.

## Introduction

Breast cancer is the most frequent type of cancer in women and one of the two leading causes of cancer deaths worldwide (Ferlay et al. 2012). The mortality rate escalates in advanced stages of the cancer where cancer cells have entered the circulation and disseminate throughout the body. This process is called metastasis and causes 90% of cancer-associated deaths. In the first step of metastasis, cancer invasion, cancer cells invade locally into the surrounding of the tumor. Cancer cells invade with two major invasion modes (or patterns): single cell migration with no or poor contact between cancer cells, and collective migration in groups of cells where cancer cells maintain their cell-cell adhesion (Friedl and Alexander 2011). One of the prominent cell-cell adhesion proteins that can affect the cancer cell invasion mode is E-cadherin (Rodriguez et al. 2012). Loss of E-cadherin is traditionally associated with enhanced cancer invasion and promoting single cell invasion (Berx and van Roy 2009), but the expression of E-cadherin can also facilitate multicellular invasion in cancers with epithelial phenotype (Friedl and Gilmour 2009). Expression of E-cadherin in breast cancer can be abolished in two ways: by genetic inactivation due to genetic aberrations (mostly due to mutations), and by gene promoter hypermethylation (Hollestelle et al. 2013). Cells from these two categories have distinct morphologies, which are also different from the cells with wild type *E-cadherin*. Breast cancer cells with *E-cadherin* mutation have a rounded morphology, a diffuse growth pattern, and invade in a multicellular organization (Khalil et al. 2017). Breast cancer cells with hypermethylated *E-cadherin* gene promoter adopt spindle like morphology and often have markers of epithelial mesenchymal transition (EMT), a global epigenetic/differentiation program through which cancer cells gain the ability to invade and metastasize (Graff et al. 1995; Lombaerts et al. 2006). Breast cancers with the wild type *E-cadherin*, on the other hand, maintain cell-cell contact and show a collective invasion pattern with an epithelial cell morphology (Cheung and Ewald 2014).

In addition to the *E-cadherin* status of the cells, the tumor microenvironment (TME) can change the invasion pattern in breast cancers (Friedl and Alexander 2011). The TME in breast cancer consists of cellular and non-cellular components which continuously interact with the tumor (Quail and Joyce 2013). Among them, the extracellular matrix (ECM) is a fibrous network of proteins that provides the invading cancer cells with both biophysical and biochemical cues. In addition, soluble gradients of chemokines or growth factors exist in the TME. These gradients together with the remodeled ECM in the TME direct cancer cells to invade, a process called chemotaxis (Roussos et al. 2011). In vitro invasion models must recapitulate essential components of the TME in order to capture the invasion mode. Conventional in vitro models used to compare the invasion of cancer cells often do not include these components. For example, conventional Transwell and wound healing assays lack the 3D environment of the ECM as well as the possibility to maintain stable biochemical gradients around the cancer cells (Van Horssen et al. 2012; Justus et al. 2014). Moreover, a systematic study to compare the three-dimensional invasion pattern of breast cancer cells is still missing from the literature. To address these shortcomings, microfluidic chips are emerging since their flexible design and laminar flow allow biologists to generate a 3D cell culture with a controlled gradient around the cells (Polacheck et al. 2013; Wu et al. 2013). It is challenging to realize these factors in an open culture system (Sleeboom et al. 2018). Most microfluidic chips use injectable hydrogels to mimic the 3D ECM. However, hydrogels have disadvantages; they offer only limited possibilities to create a well-controlled fibrous matrix structure, often show low mechanical stability over time, and do not allow retrieval from the chip for post-analysis. As an alternative to hydrogels, we have previously developed a microfabrication method to integrate engineered and mechanically more stable 3D matrices inside microfluidic chips (Eslami Amirabadi et al. 2017).

In the present study, we applied our previously developed microfabrication method to realize microfluidic chips with a thick integrated polycaprolactone (PCL) electrospun fibrous matrix, to quantitatively compare the invasion of three breast cancer cell lines with distinct *E-cadherin* status in 3D. We used a perfusion system to create a serum gradient (as a chemoattractant) around the cancer cells during the experiments. We used MCF-7, CAMA-1 and MDA-MB-231 cells with wild type *E-cadherin*, in-frame *E-cadherin* mutation and hypermethylated *E-cadherin* promoter, respectively. CAMA-1 cells do not express functional E-cadherin and MDA-MB-231 cells completely lack E-cadherin expression, whereas MCF-7 cells express functional E-cadherin. We first characterized the microfluidic system and also E-cadherin expression on the matrix. After culturing the cells inside the microfluidic chip, we found that, after 1 day, the MDA-MB-231 cells invaded more in the presence of gradient than in a positive control condition where the serum is available everywhere. After 3 days, this was inverted and the cells invaded more in the positive control. Moreover, MDA-MB-231 cells showed a uniform single cell migration pattern and invaded deeper into the matrix after 3 days compared to CAMA-1 and MCF-7. CAMA-1 cells invaded into the matrix mostly with a multicellular pattern, and showed the multifocal behavior seen in lobular breast cancers. MCF-7 cells invaded into the 3D matrix in a collective mode maintaining cell-cell contact. These results are consistent with what is generally known from the cancer biology literature (Cheung and Ewald 2014; Graff et al. 1995; Khalil et al. 2017; Lombaerts et al. 2006), and they show that our system is able to quantitatively capture the invasion ability and the invasion mode of the breast cancer cell lines in an engineered fibrous 3D microenvironment, under controlled conditions. Hence it forms a major advancement over 2D assays like the Transwell or wound healing assays, and it is a viable alternative to hydrogel-based microfluidics-based approaches, with the advantage of enabling use of stable engineered fibrous matrices.

## Results and discussion

In the following, we first characterize the microfluidic system and the cells with respect to E-cadherin, and then compare the invasion of the cells into the electrospun matrices.

### Design of the microfluidic system

**Fig. 1:**
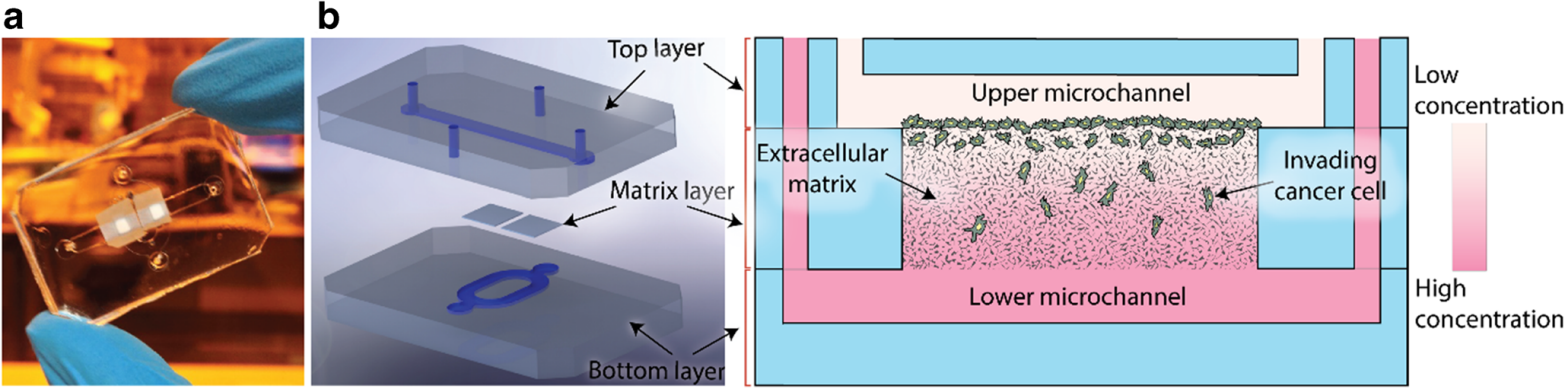
(A) Microfluidic chip used in this study, introduced in Eslami Eslami Amirabadi et al. 2017. (B) An exploded (left) and cross sectional (right) views of the tri-layer microfluidic chip; it consists of two microfluidic layers that are separated by thick electrospun matrices. The matrices are integrated in the chip using a method called selective curing. The cells are seeded on top of the matrix inside the upper (tumor) microchannel. The cells migrate into the matrix towards the bottom microchannel. The two microchannels are refreshed with different media to maintain a gradient of nutrients across the matrix layer

In order to compare the invasion of the three breast cancer cell lines (MCF-7, CAMA-1 and MDA-MB-231), we used the 3D invasion assay developed in a previous study (Eslami Amirabadi et al. 2017). The chip consisted of two polydimethylsiloxane (PDMS) microchannels on top of each other that were separated by thick electrospun polycaprolactone (PCL) matrices in two locations (Fig. 1). The matrices had a mean fiber diameter of 3.6 ± 0.3 μm and the matrix layers had an average thickness of 144 ± 14 μm (Fig. s1). PCL is an excellent choice of material when it comes to fabricating reproducible ECM matrices (Pham et al. 2006). We previously observed that breast cancer cells were able to migrate into these matrices and make 3D contact with the fibers. Here, like in Transwell assays that include thin porous polymer membranes, the cells move through a synthetic environment, however the three-dimensional fibrous matrix layer facilitates a larger migration distance as well as a 3D invasion trajectories for the cells. Furthermore, we incubated the fibrous mesh with fibronectin overnight to enhance cell adhesion and wettability of the fiber surface. In order to couple a perfusion system with the chip, we glued female luer adaptors to the inlets and outlets with viscous and semi-cured PDMS. The luers also acted as fluidic reservoirs to seed the cells in the chip with a controlled flow of the cell suspension. After seeding, the cells adhered to the bottom of the upper microchannel where they started to invade into the matrix (Fig. 1(B)). We designed a perfusion system (Fig. s2) where the medium reservoirs, connected to the inlets, stayed inside the incubator and a syringe pump, outside the incubator, withdrew the liquid from the outlets of the chips. In this way, the perfusion created a negative pressure in the chip by which bubbles were removed more easily from the fluidic circuit. The system was sealed and did not suck in any air. In addition, we only needed to add media in the reservoirs in order to refresh the media while the experiment was running. During the experiments, the bottom microchannels received culture medium with 10% serum while we exposed the cells in the upper microchannel to two conditions; in the first condition (gradient condition, Fig. 1(B)) the perfusion fed the cells with culture medium without serum, while in the second (non-gradient condition, positive control), it provided the cells with the medium containing serum (complete medium). A negative control, where the starvation medium flows to both microchannels for up to 3 days, would damage the cells and decrease cell viability (Fig. s3). Experiments with Fluorescent dextran showed that the perfusion system refreshed both microchannels with the right media (Fig. s4). We refreshed media in the microchannels every 100 min to stimulate cells with (different) media and at the same time not disturb the cells with the flow. A simulation of the concentration of growth factors in the chip is shown in Fig s4. It shows that the gradient will cease to exist after 100 min, if the media is not refreshed. More frequent refreshment than each 100 min was not used because more frequent flow could damage the cells.

After the experiments, we disconnected the chips from the perfusion system, fixed the cells and stained them for further analyses. At this point, we isolated the matrices with the cells from the chip, cryo-sectioned them and determined the invasion depth of the cells into the matrix using fluorescent imaging. We defined the invasion depth as the distance of the cells farthest from the top surface of the matrix (the starting point of the invasion). Retrieving the matrices from the chips provided the opportunity to image the invading cells with a better resolution than confocal microscopy. Furthermore, the cells and the matrix are more accessible, than injectable hydrogels, for different analytical approaches, such as secretome proteomics or mechanical testing. Reproducibility of the synthetic matrices also enables us to study the effect of different junction proteins in repeatable way.

### E-cadherin expression of breast cancer cell lines on nanofibers and microfibers

**Fig. 2:**
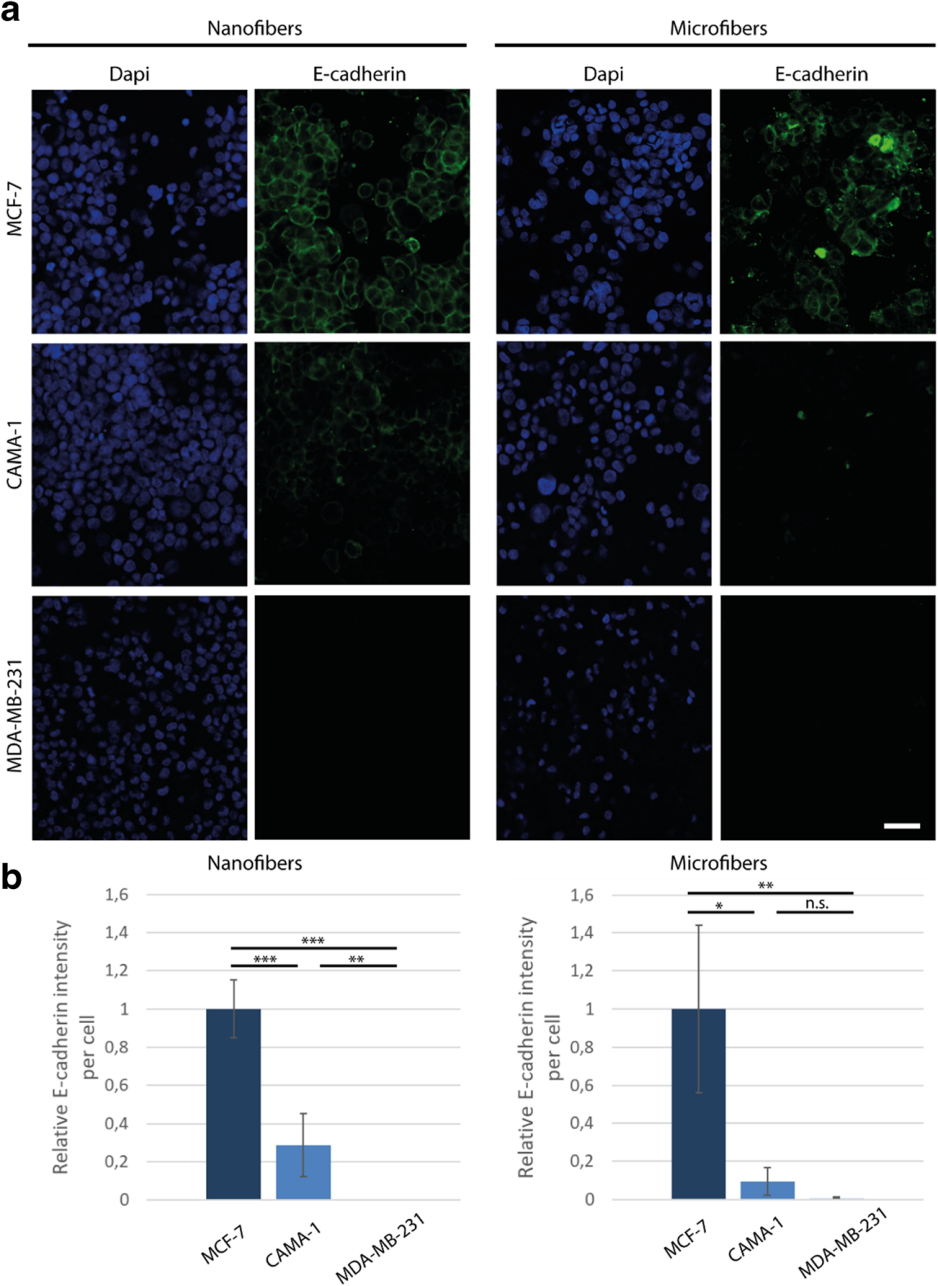
E-cadherin expression in three breast cancer cell lines on nanofibrous and microfibrous PCL matrices after 3 days. **(A)** Top view of three breast cancer cell lines on top of electrospun PCL matrices made of nanofibers and microfibers. The cells were stained with Dapi (cell nucleus, blue) and E-cadherin (green). MCF-7 cells clearly express more E-cadherin than the two other cells both on nanofibers and microfibers. The lower intensity of E-cadherin of the cells on the microfibers is because the cells invaded into the matrix and the cell-cell contact in these cells appeared in different planes. **(B)** Quantified fluorescence E-cadherin signal from the cells on top of the electrospun matrices. The intensity of the signal was measured and normalized by the number of the cells within the regions of interest. All the values were then normalized to the average intensity of E-cadherin from MCF-7 cells (relative intensity). *N* = 3 independent matrices, 2 pictures were taken from each matrix. The data were analyzed with independent sample t-test, corrected for multiple testing. n.s.: not significant, the scale bar is 50 μm; *P < 0.05, **P < 0.01, ***P < 0.001

Next, we investigated the E-cadherin expression of the breast cancer cell lines on the electrospun matrices. We cultured the cells on the matrices attached to PDMS wells for 3 days. The wells enabled us to keep the cells on the matrix at the time of seeding. To investigate whether the cells showed the same behavior as in 2D, we also seeded them on top of electrospun PCL nanofibers (with average diameter of 300 nm, Fig. s5) where they form a 2D monolayer. Figure 2 shows the measured E-cadherin expression of the cells on the nanofibrous and microfibrous matrices. As also seen in previous studies (Kenny et al. 2007; Van De Wetering et al. 2001), MCF-7 and CAMA-1 did express E-cadherin on nanofibers and microfibers although this expression seems to be significantly lower in CAMA-1 than MCF-7. MDA-MB-231 cells lacked the E-cadherin expression on both matrices. Since we have used a non-confocal microscope for these pictures, the fluorescent signals received from the cells on the microfibrous mesh are weaker. The quantitative analysis in Fig. 2(B) shows that the cells on microfibers follow a similar trend in E-cadherin expression to the cells on the nanofibers. These results conform with the expected behavior of E-cadherin expression of the cells after three days of culture on the fibrous matrices.

### Invasion of MDA-MB-231 breast cancer cells into the electrospun matrix

**Fig. 3:**
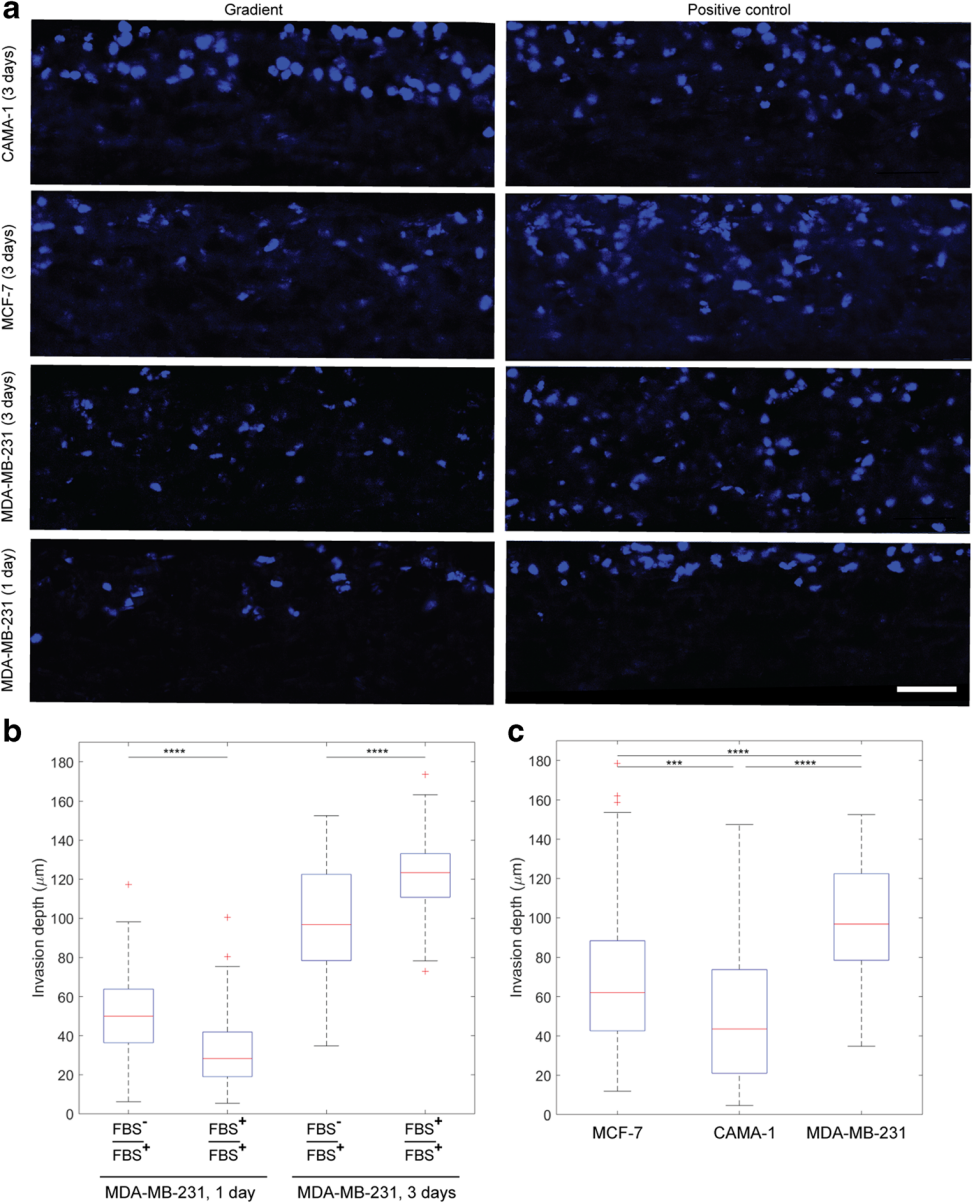
**(A)** Invasion of breast cancer cell lines into the electrospun PCL matrices in the microfluidic chips. The cross sections show the invasion of the cells under the gradient and positive control conditions. The cells are seeded on top of the matrix which is the top side of the images. The nuclei are stained with Dapi (blue). The scale bar is 50 μm. **(B)** Invasion depth of breast cancer cell line MDA-MB-231 after 1 and 3 days. The median invasion depth of the cells was 50 μm and 28 μm after 1 day and 97 μm and 123 μm after 3 days for gradient and positive control, respectively. **(C)** Invasion depth of breast cancer cell lines MCF-7, CAMA-1 and MDA-MB-231 under the gradient after 3 days. The median invasion depth for MCF-7, CAMA-1 and MDA-MB-231 cells was 62 μm, 44 μm and 97 μm, respectively. The box plots divide each distribution into four sections each containing 25% of the data. The red lines denote the medians of the distribution. *N* ≥ 3 independent matrices from at least 2 independent chips. 5 sections per matrix were imaged and the maximum invasion depth of the cells at least at 5 different locations of each section was measured (> 25 cells per matrix). The measured invasion depths (>75 cells per cell line per condition) were pooled together and a box plot of the data was created. The data were analyzed with Mann-Whitney U test. ***P < 0.001, ****P < 0.0001

We studied the invasion of MDA-MB-231 cells over time in the gradient and positive control conditions. Throughout this text, “invasion depth” indicates the net invasion depth of the cells into the matrix. The serum contains nutrients for the cells to proliferate. We seeded equal number of cells in all the conditions and ran the experiments for 1 and 3 days. Figure 3(A) shows the cross section of the electrospun matrices. After 1 day, the cells invaded more under the serum gradient than in the control (50 μm vs. 28 μm, Fig. 3(B)). We saw the same effect after 12 h in a Transwell assay (Fig. s6). However, after 3 days, the cells invaded more in the positive control than the gradient condition (123 μm vs. 97 μm). We observed more cells in the sections in the positive control which is probably because they received enough nutrients present in the complete medium to maintain the proliferation, whereas in the gradient condition, the cells received limited nutrients only by diffusion from the bottom channel. Culturing cells in the medium without serum indeed confirmed lower cell proliferation and cell viability (Fig. s3). We speculate that the serum gradient can drive the invasion in a short run, for example in our case after 24 h, but at a larger time scale, e.g. 3 days, lack of nutrients hinders cancer cell proliferation and invasion in the gradient condition. The doubling time of the MDA-MB-231 cells is around 25 h (Mason et al. 2014) in the normal serum condition which means that after this time, the increased cell density enhances the invasion. Indeed higher cell density has been reported to override other environmental factors during the invasion of MDA-MB-231 cells (Polacheck et al. 2011). Conventional chemotaxis assays, such as Transwell assay, are not able to capture this behavior since they do not control medium content in different compartments over time. Therefore, the current system can be a good alternative to the conventional assays especially where the experiments require to isolate the cells and matrix from the system without dissociation of the sample.

The MCF-7 and CAMA-1 cells did not show significant invasion in the matrix after 1 day, and therefore only data after 3 days of invasion are reported for these cells, in the next section. The doubling times of MCF-7 and CAMA-1 cells are 51 and 70 h, respectively (Mason et al. 2014).

### Comparing the invasion of breast cancer cell lines with different *E-cadherin* status

Next, we used the microfluidic system to compare the invasion ability of three breast cancer cell lines MCF-7, CAMA-1 and MDA-MB-231 in a 3D environment in the gradient and positive control conditions. The cell lines have different *E-cadherin* status and morphology (Riaz et al. 2013). MCF-7 cells grow in epithelial sheets and migrate collectively. They are wild type for *E-cadherin*. CAMA-1 cells are mutant for *E-cadherin* and show rounded morphology. These cells are from the lobular type of breast cancer that invade in a multicellular pattern as strands, cluster or Indian files(Khalil et al. 2017). MDA-MB-231 cells have spindle-like cell morphology, lack E-cadherin expression due to hypermethylation of the gene promoter and usually invade as single cells. In 2D, the invasion distance of all of the three cell lines have been compared in a wound healing assay (Van Horssen et al. 2012).

Figure 3 shows the invasion of the cell lines in the gradient condition. Highly invasive MDA-MB-231 cells had the largest invasion depth. Under the serum gradient, MCF-7 cells invaded more than CAMA-1 cells but this difference was not significant under the positive control condition (Fig. s7). We observed more migration in the positive control for CAMA-1 cells compared to the gradient condition (Fig. s7), hence showing the same behavior as MDA-MB-231 cells after 3 days (Fig. 3(B)). As mentioned before, both MDA-MB-231 and CAMA-1 cells do not express (functional) E-cadherin. Comparing the invasion depths of these cells suggests that the mode of *E-cadherin* inactivation, rather than inactive E-cadherin, is part of the events that determine the invasiveness of the cells. In addition, invasion of MCF-7 cells showed that E-cadherin did not suppress the invasion in this breast cancer cell line.

**Fig. 4:**
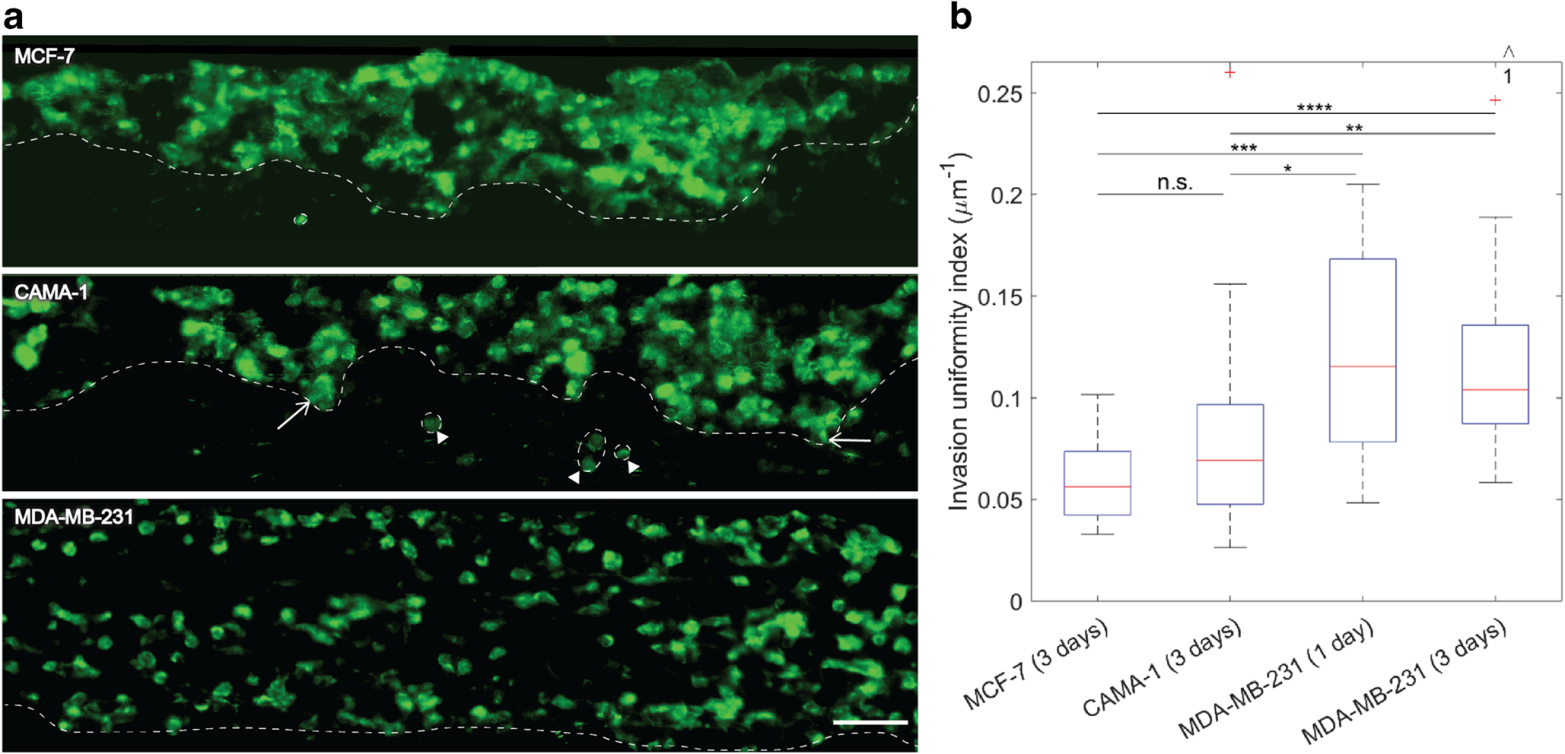
**(A)** Invasion of breast cancer cell lines into the electrospun PCL matrices inside the microfluidic chip. The cross sections show the invading cells under the positive control condition after 3 days. The cells (green) were seeded on top of the matrix which is the top side of the images. The cells invaded into the matrix individually (MDA-MB-231) or in clusters (CAMA-1 and MCF-7). The open arrows show the clusters of CAMA-1 cells, and the arrow heads show smaller cell clusters near the invading front line indicating the multifocal behavior of the cells. The white dashed lines show the invading front of the invading cells, indicating how uniform the cells invade to the matrix. The cells were stained with Phalloidin Atto 488 (cytoskeleton). The scale bar is 50 μm. **(B)** The invasion uniformity index, defined as the inverse of standard deviation of the invasion depths measured in each section of the matrix. Higher uniformity index corresponds to a more uniform invasion pattern. MDA-MB-231 cells invaded to the matrix uniformly across the matrix and therefore had higher invasion uniformity index, with a median of 0.1153 μm^−1^ and 0.1040 μm^−1^ for 1 day and 3 days, respectively. On the other hand, CAMA-1 and MCF-7 cells invaded to the matrix in a non-uniform way. Median invasion depths for CAMA-1 and MCF-7 cells are 0.0692 μm^−1^ and 0.0561 μm^−1^, respectively. *N* ≥ 3 independent matrices from at least 2 independent chips. The data were analyzed with Mann-Whitney U test. *P < 0.05, **P < 0.01, ***P < 0.001, ****P < 0.0001, n.s. indicates no significance. The arrows and numbers on top of the graphs demonstrate the number of outliers outside the range of the y axis

The values of invasion depth in Fig. 3(C) are lower than in two dimensional experiments (Van Horssen et al. 2012). A main reason for this difference may be that the 3D matrix presents the cells with geometrical confinement which hinders the invasion of the cells. Also, cell adhesion to 2D substrates could differ from those in our matrix. However, the order of invasion is the same as what we see in 2D, meaning that MDA-MB-231 cells invade more than MCF-7 cells and MCF-7 cells invade more than CAMA-1 cells. Also, the matrix remodeling, e.g. proteolysis activity of the cells is ineffective in the synthetic PCL matrix, which may be a reason for less invasion of the cells compared to other 3D models with natural hydrogels as the ECM (Kumar et al. 2016; Truong et al. 2016). The advantage of the chip used in this study was that we could integrate matrices other than hydrogels. i.e. engineered fibrous matrix layers, and that we were able to retrieve the matrix from the chip intact. In future experiments, also self-standing natural matrices can be integrated in the microfluidic chip, such as electrospun collagen matrices. Moreover, we compared the mode of invasion of the three different cell lines. MDA-MB-231 cells do not express cell-cell junctions, such as E-cadherin, and invade as single cells or as multicellular streams without cell-cell adhesion proteins (Patsialou et al. 2013). We found that these cells invaded the PCL matrix as single cells in a uniform pattern independent of the location, as shown in Fig. 4(A). CAMA-1 cells are mutant for E-cadherin and show rounded morphology in 2D. In addition to a diffuse growth pattern, a characteristic of lobular breast cancers (Hollestelle et al. 2010), they show a multifocal behavior (Christgen et al. 2016). When tested inside the microfluidic chip in the 3D PCL matrix, they mostly kept the multicellular invasion mode, mainly as tumor nests or clusters (open arrows in Fig. 4(A)). Interestingly, they also showed the multifocal invasion with small cell clusters close to the invading front (arrowheads in Fig. 4(A)) less uniformly than MDA-MB-231 cells. MCF-7 cells are epithelial like and wild type for *E-cadherin*. They mostly invade collectively in 2D and maintain cell-cell contact during invasion (Mendoz and Lim 2011; Planas-Silva and Waltz 2007). As seen in Fig. 4(A), these cells migrated into the matrix mostly in a multicellular non-uniform way.

To quantify the uniformity of invasion, we defined an invasion uniformity index (Fig. 4(B)). As explained in the section “Materials and Methods”, we measured the invasion depth in 5 different sections of each matrix located at least 60 μm apart from each other. We calculated the standard deviation of invasion depth in each section and defined the invasion uniformity index of the section as the inverse of this standard deviation. The higher uniformity index for MDA-MB-231 cells in Fig. 4(B) means that these cells invaded through the matrix more uniformly (lower standard deviation of invasion depth). In contrast, the lower uniformity index for CAMA-1 and MCF-7 suggests that these cells adopted a local non-uniform invasion pattern (higher standard deviation of invasion depth). Non-uniform invasion seems to be connected to the multicellular invasion. This may be because cells in groups tend to choose larger pore sizes to invade into the PCL matrix that has a wide distribution of pore size (see Fig. s1). This preference decreases the number of paths through which the cells can infiltrate into the matrix and therefore makes the invasion a local event.

In summary, we were able to quantitatively compare the invasion depth and invasion mode of the cells in our microfluidic system.

## Materials and methods

### Microfabrication methods: Electrospun matrix and microfluidic chip

The reader is referred to the previous study for the details of the microfabrication processes(Eslami Amirabadi et al. 2017). Briefly, a solution of 15% Poly caprolactone (Corbion Purac Biomaterials, the Netherlands) in 1,1,1,3,3,3-Hexafluoro-2-propanol (HFIP, Sigma Aldrich, the Netherlands) for microfibers and a solution of 12% PCL in Chloroform: Methanol (5:1) for nanofibers was made. These were electrospun using an EC-CLI electrospinning apparatus, IME Technologies, to obtain fibrous matrix layers with average thickness of 144 ± 14 μm. Occasionally, random spikes of fibers come out of the fibrous mesh which locally increases the thickness of the mesh. However, as shown in Fig. s1(C), these are usually outliers and therefore do not affect the results. For fiber thickness distribution, we chose 5 images per condition and measured the average thickness of the fibrous mesh in each image. The average diameter of the microfibers and nanofibers were 3.6 μm and 300 nm, respectively.

For evaluating E-cadherin expression on the microfibers and nanofibers, blank PDMS (Sylgrad 184, Dow corning, Germany) was partially cured at the ratio of 1:10. Cubes of approximately 10*10*4 mm were cut out and punched with an 8 mm puncher (PDMS well). Matrices, either nanofibrous or microfibrous, were cut to approximately 8*8 mm and sandwiched between two PDMS wells. The sandwich was left at room temperature to cure completely.

The microfluidic chip consisted of two layers of microchannels made of PDMS with two PCL electrospun matrices in between, as seen in Fig. 1. Soft lithography was used to make the microchannels. The microchannels were approximately 400 μm high, 2.5 mm wide and 25 mm long. The matrices were integrated in the chip using a method called selective curing (Eslami Amirabadi et al. 2017). Briefly, liquid PDMS without curing agent was spin-coated on a microscope slide and two pieces of the matrix were applied in the PDMS film on the microscope slide. PDMS with curing agent (ratio of 1:10) with the microchannel features was partially cured and aligned with the matrices and gently left on the microscope slides. The assembly was left overnight to cure completely at room temperature. A second PDMS layer was partially cured and bonded to the first layer with the same method. Now, the PDMS without curing agent inside the matrix was cured, by diffusion of curing agent from the microchannel layers, everywhere except where the microchannels crossed each other since the matrix did not make contact with PDMS parts at that location. PDMS in this location was subsequently removed by flushing the chip with Isopropanol two times with a time difference of at least 4 h. The chip was then washed with distilled water.

Using our previous publication (Eslami Amirabadi et al. 2017), we assumed that the diffusion coefficient of small growth factors (such as EGF) is D_matrix_ = 0.1 × D_water_, where D_water_ is the diffusion coefficient of the growth factors in an aqueous solution or medium. COMSOL multiphysics was used to simulate the diffusion of the growth factors inside the matrix, see Fig. s4. For simplicity, a concentration of 0 mol/m^3^ and 1 mol/m^3^ were assumed in the top and bottom microchannels, respectively. Changing the concentration in the chip does not change the diffusion profile in time.

### Preparation of the microfluidic chips

PBS (Lonza, Westburg, the Netherlands) was injected into the chips and the chips were degassed for 15–30 min. New PBS was injected into the chips to completely fill the microchannels and the luer connections acting as reservoirs near the inlets and outlets. All surfaces of the chips were sterilized with 70% ethanol (*v*/v). The liquid exchange inside the microchannels was generated by a height difference between inlets and outlets. After washing the ethanol with PBS, the microchannels as well as the electrospun matrix were coated with 50 μg/ml fibronectin (Merck Chemicals, the Netherlands) in PBS overnight at 37 °C. The fibronectin solution was subsequently replaced by starvation medium.

### General cell culturing

MDA-MB-231 and CAMA-1 breast cancer cell lines are described in Hollestelle et al.(Hollestelle et al. 2007). MCF-7 cells were purchased from Sigma Aldrich, the Netherlands. The cell lines were maintained and expanded in RPMI-1640 Glutamax medium (Gibco, Thermofisher Scientific, The Netherlands) supplemented with 10% (v/v) foetal bovine serum (FBS, Bovogen Biologicals, Australia and Lonza, Breda, the Netherlands) and 1% Penicillin-Streptomycin (P/S, Lonza, Westburg, the Netherlands). The starvation medium used was RPMI-1640 Glutamax medium (Gibco, Thermofisher Scientific, The Netherlands) supplemented with 0.1% (*w*/*v*) bovine serum albumin (BSA, Roche diagnostics, Indianapolis, USA) and 1% P/S (Lonza, Westburg, the Netherlands). In 70% to 90% confluency, the cells were removed from the surface by trypsin EDTA (Lonza, Westburg, the Netherlands), centrifuged at 900 rpm for 5 min, and resuspended in either normal (to culture in a flask) or starvation medium (to seed the chips).

To test the expression of E-cadherin on the nanofibrous and microfibrous matrices, these matrices were first sterilized with 70% ethanol in water, washed with PBS and then coated with 50 μg/ml fibronectin (Merck Millipore, the Netherlands) overnight in a wells plate. After removing the fibronectin, the wells and the matrices were hydrated in PBS and saved in a fridge for cell culturing. 20 μl of the cell suspension with a density of 6 × 10^6^ cells/ml was put on the matrices. After 2 h, the cells were supplied with fresh medium and maintained at 37 °C and 5% CO2 for 4 days.

To test the viability of the cells with the normal and starvation media, we seeded 0.2 × 10^6^ cells/ml to a 12-wells plate with the two media and maintained the culture at 37 °C and 5% CO_2_ for 3 days.

### Invasion assay

The cells were starved with the starvation medium overnight. After removing from the surface and centrifuging, the cells were resuspended in the starvation medium. They were then counted using NucleoCounter NC200 (Chemometec, Denmark) and diluted to obtain a concentration of ~ 8.5 × 10^6^ cells/ml. The reservoirs were filled with medium, and the ones connected to the inlet and outlet of the bottom microchannels were blocked using luer plugs. The other reservoirs were then emptied. 100 μl of the cell suspension was added to the inlet of the upper microchannel to create a flow to the outlet. After at least 4 h of incubation, the chips were connected to the perfusion system to automatically refresh the media (Fig. s2). Refreshing the medium is necessary for creating and maintaining the chemotactic gradient. A syringe pump (Nexus 3000, Chemyx, Texas, USA) was used for this purpose. A gentle flow (50 μl/min) was created for 1 min every 100 min. As shown in Eslami Amirabadi et al. 2017 and Fig. s4, this refreshes the gradient while not disturbing cells with the shear stress from the flow. Both microchannels of the control chips were refreshed with normal medium. The top and bottom microchannels of the chemotaxis chip were refreshed with starvation and normal medium, respectively.

For the Transwell invasion assay, the wells and inserts, in a 24 well format with a pore size of 8 mm (Corning Transwell, Sigma Aldrich the Netherlands), were coated with 50 μg/ml fibronectin overnight. Normal medium was added to all the wells. The cells were resuspended in the normal (for positive control) and starvation (for gradient) media and 100 μl of approximately 1 × 10^6^ cells/ml cell suspension was added to the inserts. The experiments lasted 12 h.

### Staining and cryosectioning

After running the invasion assays, the cells were fixated using 3.7% paraformaldehyde (Sigma Aldrich, the Netherlands) for 30 min. The matrices were isolated from the chips and washed with 0.5% Triton X-100 for 15 min, after which they were washed with PBS. The cells were stained with DAPI (1:500 dilution, Sigma Aldrich, the Netherlands) and Phalloidin Atto 488 (Sigma Aldrich, the Netherlands) to visualize the nuclei and the actin cytoskeleton of the cells, respectively.

The stained matrices were embedded in TissueTEK (Sakura Finetek, the Netherlands). To freeze the samples, 10 ml Iso-pentane (Merck Chemicals, the Netherlands) was added to a beaker and was left on dry ice or liquid nitrogen for at least 5 min to ensure Iso-pentane was cooled down. Then, the samples were placed in the beaker until it was completely frozen. The samples were stored for at most one week at −30 °C. Using a cryotome (Microm HM550 cryostat microtome, Thermo Scientific, the Netherlands) sections with a thickness of 20 μm were made at −20 °C and attached to microscope slides (Polysine slides, Thermo Scientific, the Netherlands). This protocol was optimized for sectioning quality, minimal deformation and motion of matrix fibers and cells, see (Eslami Amirabadi et al. 2017).

For the visualization of the E-cadherin in the cells on both nanofibers and microfibers, the cells were fixed using 3.7% paraformaldehyde (Sigma Aldrich, the Netherlands), permeabilized with 0.5% Triton X-100 for 10 min and subsequently washed with PBS. Then, the constructs were blocked using 4% goat serum in 0.05% Tween-PBS for 30 min, after which E-cadherin monoclonal primary antibody (HECD-1, 5 μg/ml, Thermofisher Scientific, the Netherlands) was incubated overnight at 4 °C. The next day, the matrices were washed with PBS and a secondory antibody (1:500 dilution, Alexa Fluor 488 goat anti-mouse IgG1, Thermofisher Scientific, the Netherlands) was added to the samples for 1 h. In order to relate the expression to the amount of cells present, the cells were again stained with DAPI (1:500 dilution, Sigma Aldrich, the Netherlands)).

Viability of the cells was tested using the ReadyProbes cell viability imaging kit (Life Technologies, USA). Viability was tested after 3 days of culturing cells in the normal and starvation media in a wells plate. 2 drops of each probe were added to 1 ml of medium and then added to the wells. The cells were incubated for 15 min and then imaged for blue (alive) and green (dead) with an EVOS FL cell imaging microscope (ThermoFisher scientific, the Netherlands).

### Microscopy

The fluorescent imaging of the E-cadherin expression and invasion was performed with a Zeiss LSM510 META NLO (Zeiss Nederland, the Netherlands) with a 20x objective. PCL is autofluorescent with low intensities in all wavelengths. No fluorescence filter was used to visualize the Autofluorescence of the PCL matrix in order to have an indication of the boundaries of the matrix. 5 sections per matrix, at least 60 μm apart, were imaged.

To generate a tiled image, the mosaic function of a Zeiss Axiovert 200 M microscope with a blue filter (Dapi staining) with a 10x objective was used to image the cells attached to the membrane of the Transwell inserts.

### Data analysis

All images were analyzed using ImageJ. The data for each category (per cell line and per condition) was obtained from more than three independent matrices from at least two independent chips. Five sections per matrix were imaged and the maximum invasion depth of the cells at least at 5 different locations of each section was measured (more than 25 cells per matrix). The measured invasion depths (more than 75 cells per cell line per condition) were pooled together and a box plot of the data was created. Matlab was used to plot all the data and the Mann-Whitney U test was used to assess the statistical significance of the differences between the data.

To quantify E-cadherin, 3 independent experiments were done. From each matrix, at least 2 sets of pictures were made from different locations. Each set of pictures contained at least one image of the E-cadherin antibody staining and one image of DAPI staining of the exact same location. The intensity of E-cadherin staining was measured and normalized to the amount of cells present. An independent sample T-test with Bonferroni correction was used to assess the statistical difference in the intensity of the E-cadherin staining.

## Conclusion

We used a previously developed microfabrication method to realize a microfluidic system that we applied to quantitatively study the invasion ability and invasion mode of breast cancer cells, with different *E-cadherin* status, while they invaded into an engineered 3D fibrous matrix under a controlled serum gradient. We cultured MCF-7 with epithelial-like, CAMA-1 with rounded (lobular) and highly invasive MDA-MB-231 cells with mesenchymal phenotype inside the microfluidic chip. We confirmed that MDA-MB-231 cells did not express E-cadherin on the matrix while the two other cell lines expressed E-cadherin although in different levels. We saw that in 3 days, MDA-MB-231 cells invaded deeper into the 3D matrix than the other two cell lines in a serum gradient. Also, both CAMA-1 and MCF-7 showed a multicellular invasion pattern while MDA-MB-231 cells invaded as single cells. We were able to capture the multicellular multifocal and multicellular collective behavior of the infiltrating CAMA-1 and MCF-7 cells, respectively. Moreover, we found that MDA-MB-231 cells invaded more in the presence of the gradient after 1 day, but migrated more after 3 days when they were supplied enough serum in the medium in both microchannels.

The results show that our microfluidic system enables the quantitative analysis of three-dimensional cancer cell invasion in an engineered 3D matrix while maintaining stable biochemical conditions, which forms a major step compared to 2D methods like Transwell of wound healing assays. Also, our study underlines that our microfluidic system provides an alternative to current microfluidic models for cancer invasion under a controlled gradient. First, the chip enables us to use other ECM models than hydrogels which offer limited possibilities to create a well-controlled fibrous matrix structure, with a characteristic of the engineered synthetic PCL matrix layer. Second, the fabrication method allows us to retrieve the tested matrix from the chip without cell or matrix dissociation. This feature opens up the possibility to utilize a diversity of analytical read-outs, such as proteomic approaches or mechanical measurements. The current investigation was a proof of concept to study the non-proteolytic invasion of cancer cells, using engineered fibrous PCL as a synthetic 3D matrix, hence the biological context of the extracellular matrix is partially not captured in the results presented. Integration of natural matrices in the chip, for example decellularized or electrospun collagen matrices (Sell et al. 2010), in the future will allow us to compare the invasion ability and invasion mode of the cells when matrix remodeling by proteolysis is present. Furthermore, other elements of the TME can be added to the chip to understand their effect on cancer invasion. For example, fibroblasts or endothelial cells can be cultured on the bottom side of the matrix to study how these cells affect cancer cell invasion speed or directionality. Finally, the effect of cell treatments on their invasion potential can be quantitatively assessed in the future.

## Electronic supplementary material

ESM 1 (DOCX 11859 kb)
