# Supplementary material for: Characterizing the invasion of different breast cancer cell lines with distinct E-cadherin status in 3D using a microfluidic system
Source: Biomed Microdevices. 2019 Nov 23;21(4):101. doi: 10.1007/s10544-019-0450-5 (PMC6875428; doi:10.1007/s10544-019-0450-5)
Supplement: Supplementary file 1 — (DOCX 11859 kb) [file 10544_2019_450_MOESM1_ESM.docx]

**Characterizing the invasion of different breast cancer cell lines with distinct *E-cadherin* status in 3D using a microfluidic system**

**H. Eslami Amirabadi,^ab^* M. Tuerlings,^ac^* A. Hollestelle,^d^ S. SahebAli,^a^ R. Luttge,^a^ C.C. van Donkelaar,^c^ J.W.M. Martens^d^ and J.M.J. den Toonder^a^†**

Supplementary information


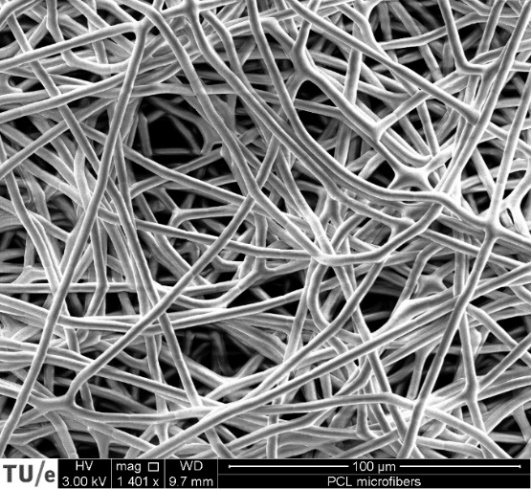

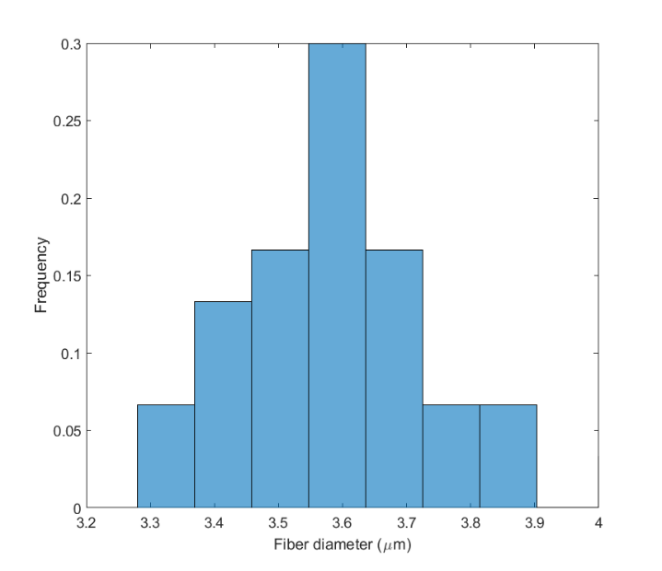


(A)

(B)

**Fig s*1* (A)** An electron scanning micrograph of the Poly caprolactone (PCL) matrix used in this study. **(B)** A histogram distribution of the fiber diameter. The mean fiber diameter is 3.6 µm with standard deviation of 0.3 µm. **(C)** A scatter plot showing the distribution of the matrix thickness. The mean thickness was 144 µm with a standard deviation of 14 µm. 5 images per condition were used in this analysis.


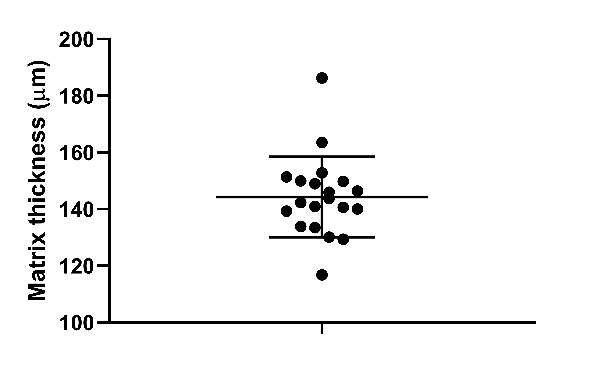


(C)


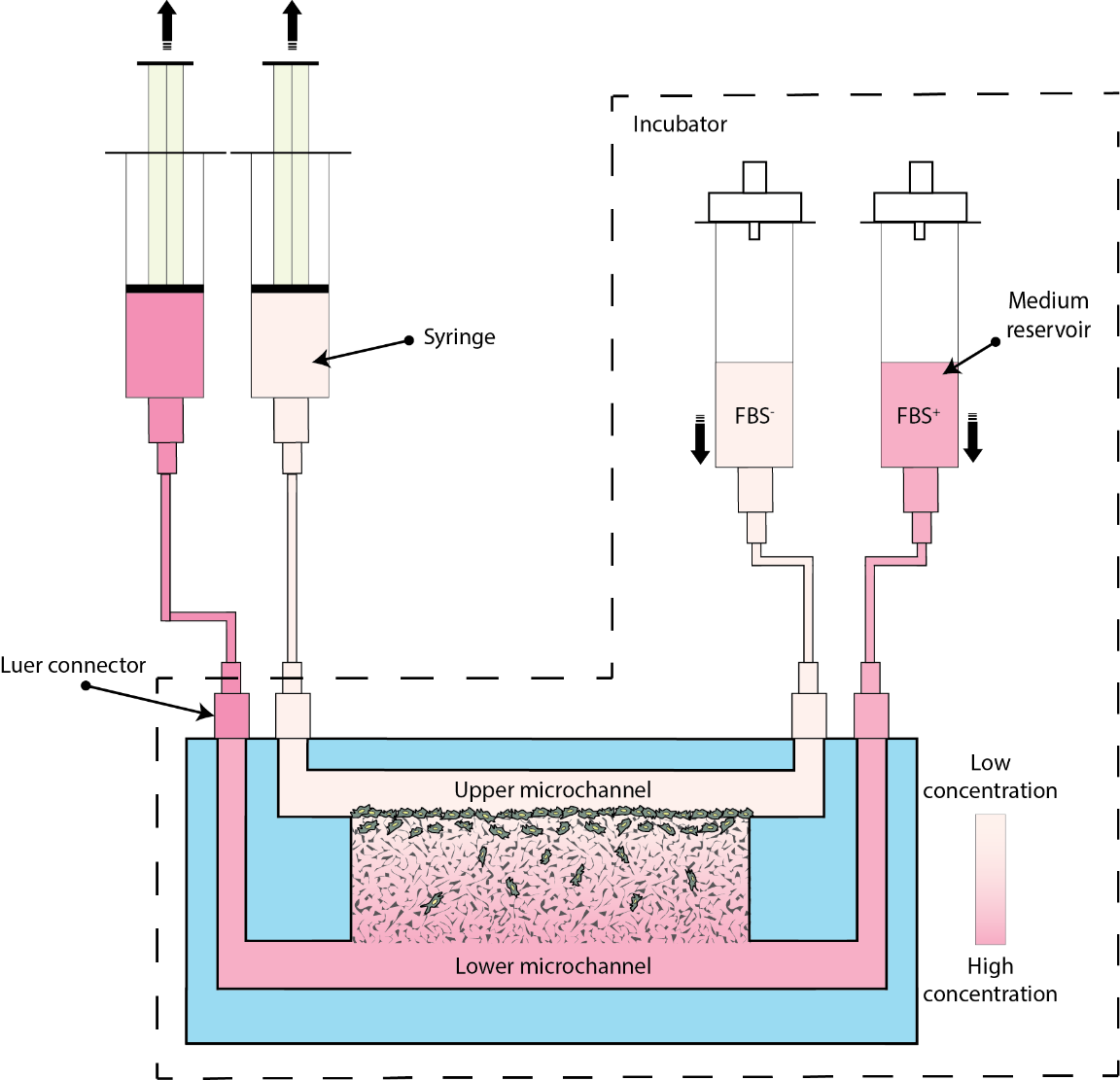


**Fig s2** Schematic of the automatic perfusion system used in this study for the chemotaxis condition. The dashed line indicates the part of the system that is inside the incubator. The inlets of the chip (right) are connected to the reservoirs which are in contact with humid air with 5% CO_2_ at 37°C. The outlets of the chip are connected to the exhaust syringes outside the incubator. A syringe pump withdraws 50 µl of the medium from each microchannel at the flow rate of 50 µl/min every 100 minutes. In control experiments, the medium with serum (FBS^+^) is used instead of the medium without serum (FBS^-^).


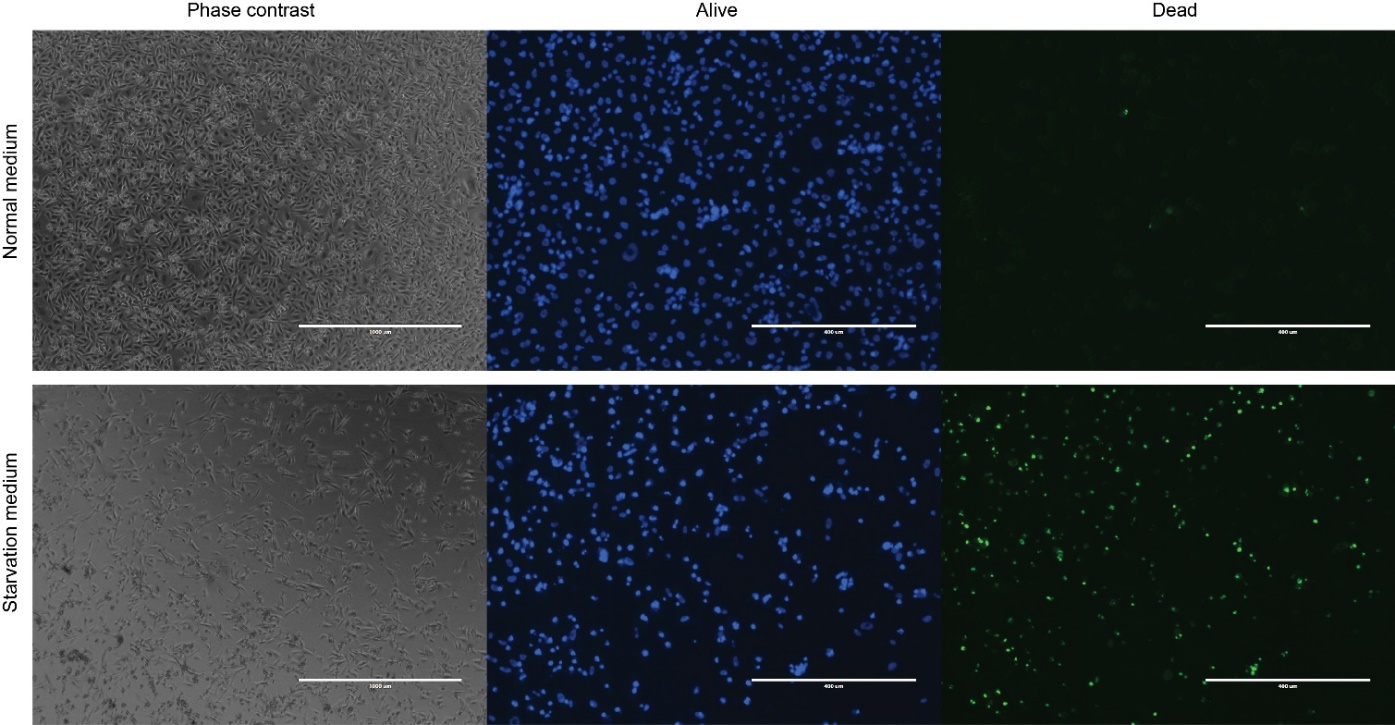


**Fig s3** Viability of MDA-MB-231 cells cultured in normal and starvation media after 3 days. The cells were seeded with a cell density of 0.2e6 cells/ml into a 12-wells plate with the normal or starvation medium. Almost no proliferation was seen in the cells with the starvation medium. Blue and green show the nuclei of the alive and dead cells, respectively. The phase contrast images were taken at different fields of view than the viability images. The scale bars are 400 µm.


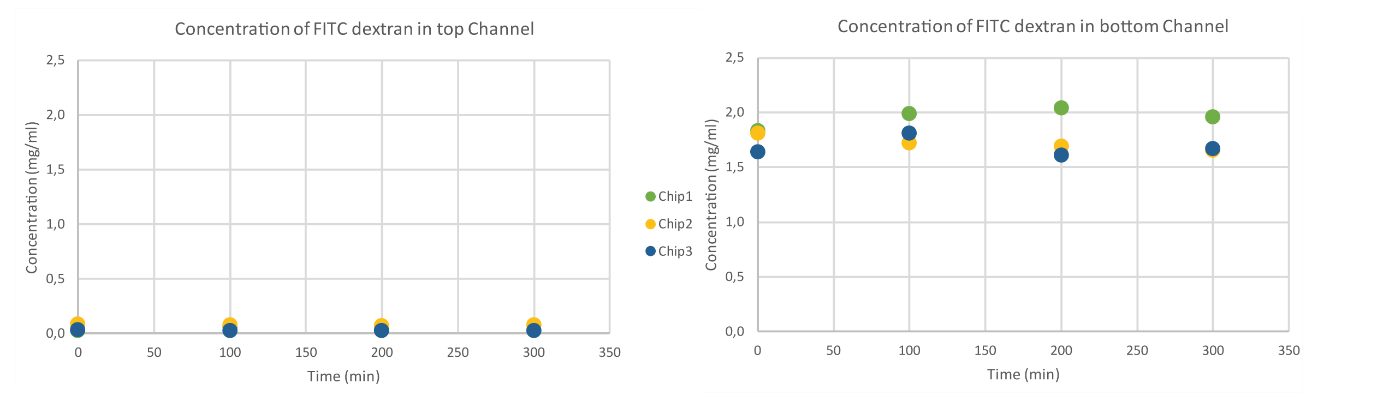


**Fig s4** **(A)** Measured concentration of a 10 kDa FITC dextran in 3 chips as a representation for the growth factors and other nutrients in the serum immediately after refreshing. The edge of one of the membranes was chosen to represent the concentration of the dextran in the whole channel. The top microchannels were refreshed with PBS and the bottom microchannels were fed with PBS + 2 mg/ml FITC dextran. The microchannels were refreshed every 100 min. **(B)** Simulation of the concentration of the fluorescent dextran on top (green line) and bottom (blue line) of the membrane during the 100 minutes. Diffusion coefficient of the GF molecules was assumed to be 0.1 times the diffusion coefficient of the same molecules in medium. For simplicity, an initial concentration of 0 mol/m^3^ and 1 mol/m^3^ were assumed in the top and bottom microchannels, respectively. Changing the concentration in the chip does not change the diffusion profile in time. **(C)** Concentration of the fluorescent dextran inside the microfluidic channels and also the membrane. For simplicity, a concentration of 0 mol/m^3^ and 1 mol/m^3^ were assumed in the top and bottom microchannels, respectively. Changing the concentration in the chip does not change the diffusion profile in time.

(A)

(B)


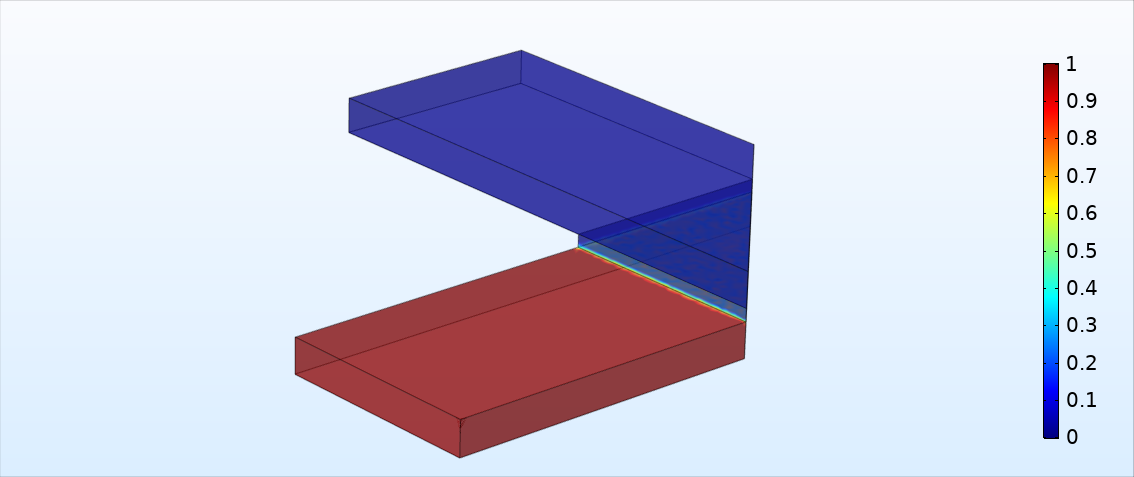

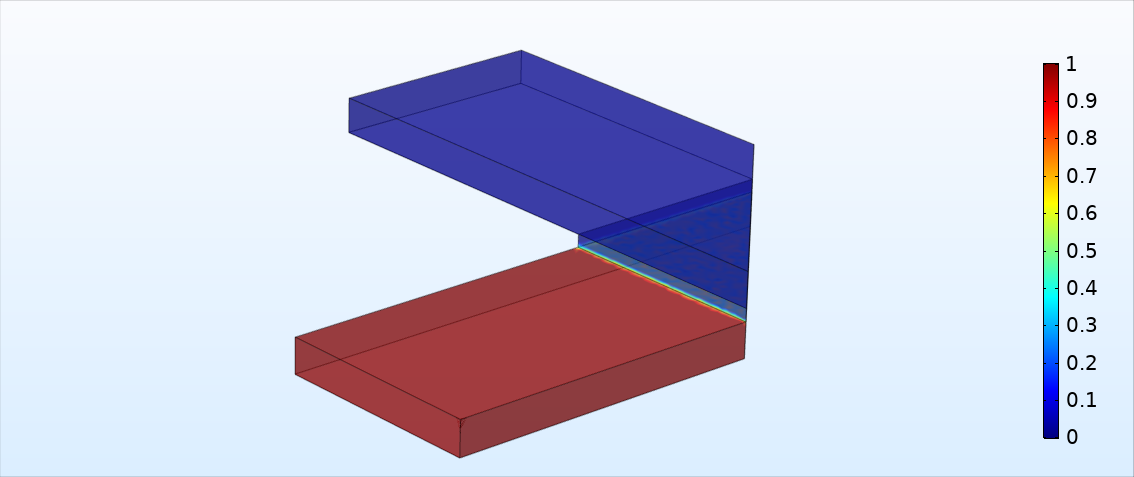

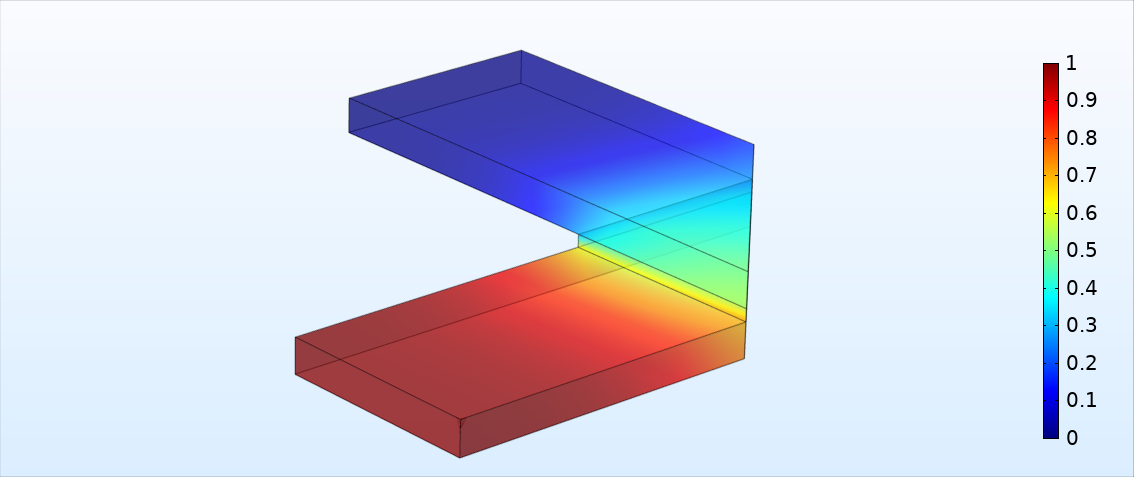


(C)

T = 0 min

T = 100 min


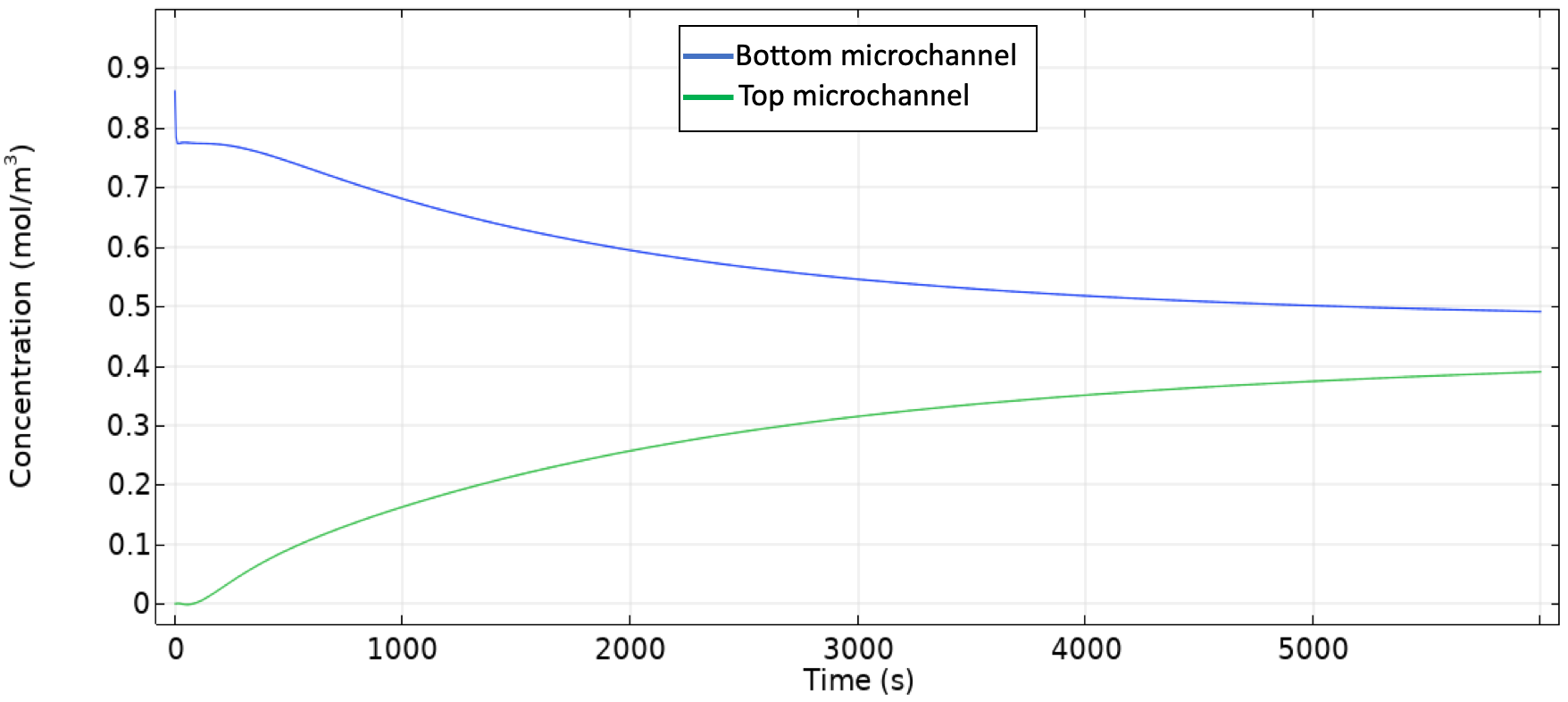


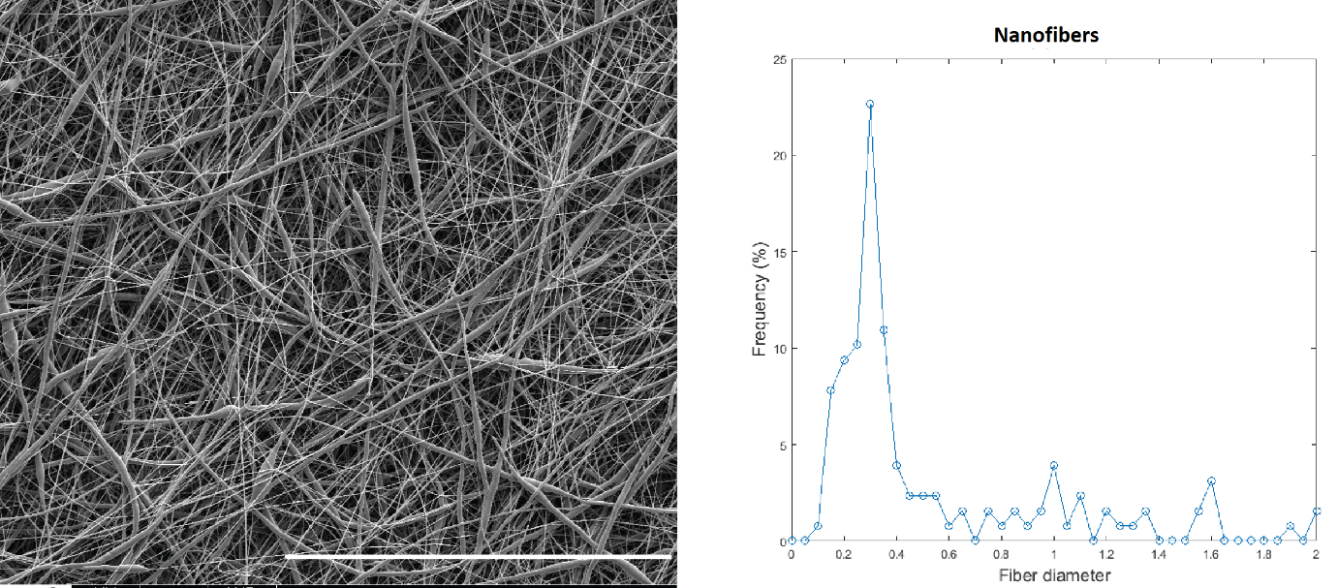


**Fig s5** Nanofiber PCL matrix with the average diameter of 300 nm. The scale bar is 100 µm.


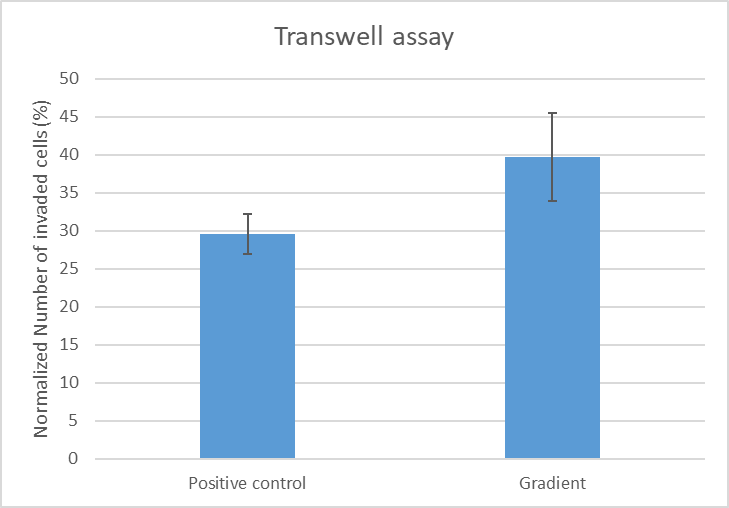


**Fig s6** Transwell invasion assay on MDA-MB-231 breast cancer cells for 12 hours. The number of the invaded cells (on the bottom of side of the Transwell membrane) was normalized with the total number of the cells (on the top and bottom of the membrane). First, the inserts were imaged using mosaic imaging and then the cells were counted using Nucleus Counter function in ImageJ. The cells on top of the insert were wiped away and the same imaging and counting were repeated for the remaining cells. N=3 and the error bars show the standard deviations.


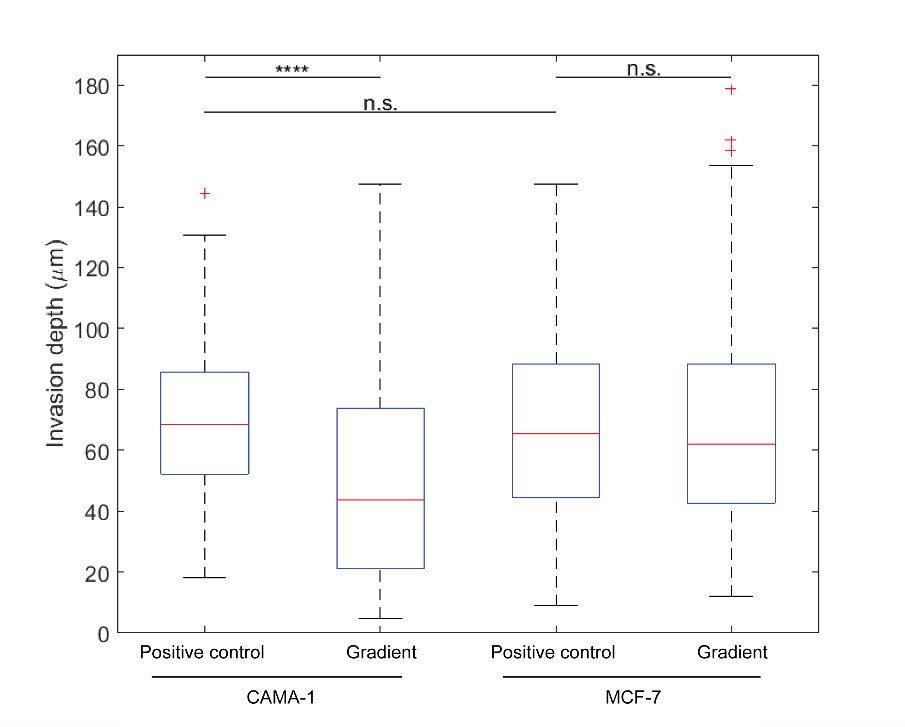


**Fig s7** - Invasion depth of breast cancer cell lines CAMA-1 and MCF-7 under control and chemotaxis conditions after 3 days. The median invasion depths of CAMA-1 cells are 68 µm and 44 µm for control and chemotaxis, respectively. The median invasion depths of MCF-7 cells are 65 µm and 62 µm for control and chemotaxis, respectively. The box plots divide each distribution into four sections each containing 25% of the data. The red lines demonstrate the medians of the distribution. The data for each category is from more than 3 independent matrices from at least 2 independent chips. 5 sections per matrix were imaged and the maximum invasion depth of the cells at least at 5 different locations of each section was measured (> 25 cells per matrix). The measured invasion depths (>75 cells per cell line per condition) were pooled together and a box plot of the data was created. The data were analyzed with Mann-Whitney U test. ****P<0.0001, n.s. indicates no significance.
